# Cardiometabolic multimorbidity prevalence and its socio-demographic determinants among Iranian adults: insights from the nationwide STEPS 2021

**DOI:** 10.1038/s41598-026-52569-6

**Published:** 2026-06-01

**Authors:** Mohammad Rahmanian, Danial Molavizadeh, Samaneh Akbarpour, Niloofar Deravi, Samaneh Asgari, Farzad Hadaegh

**Affiliations:** 1 Non-Communicable Diseases Research Center, Endocrinology and Metabolism Population Sciences Institute, Tehran University of Medical Sciences, Tehran, Iran; 2 Prevention of Metabolic Disorders Research Center, Research Institute for Metabolic and Obesity Disorders, Research Institute for Endocrine Sciences, Shahid Beheshti University of Medical Sciences, Tehran, Iran; 3 Student Research Committee, School of Medicine, Shahid Beheshti University of Medical Sciences, Tehran, Iran; 4 Prevention of Metabolic Disorders Research Center, Research Institute for Metabolic and Obesity Disorders, Research Institute for Endocrine Sciences, Shahid Beheshti University of Medical Sciences, P.O. Box 19395-4763, Tehran, Islamic Republic of Iran

**Keywords:** Multimorbidity, Cardiometabolic, Hypertension, Diabetes, Cardiovascular diseases, Socio-demographic factors, Cardiology, Diseases, Endocrinology, Health care, Medical research, Risk factors

## Abstract

Cardiometabolic multimorbidity—typically defined as the presence of two or more cardiometabolic morbidities simultaneously—has emerged as a critical public health challenge both globally and in Iran. We analyzed data from the 2021 WHO STEPwise approach to NCD risk factor surveillance (STEPS) to determine its national prevalence and associated socio-demographic factors. Cardiometabolic multimorbidity was defined as having ≥ 2 of: hypertension (HTN), type 2 diabetes (T2DM), or history of MI/stroke. Multivariable stepwise logistic regression was employed to identify the potential socio-demographic associated factors. Of total 16,453 participants aged 25–75 years (75.43% from urban areas, 54.67% women), 58.01% (confidence interval (CI): 56.92–59.09) had no morbidity, 27.56% (26.59–28.55) had only one morbidity (HTN: 19.78%, T2DM: 6.02%, MI/Stroke: 1.76%), 11.73% (11.07–12.45) had two morbidities, and 2.68% (2.31–3.11) had all three morbidities. In total, the prevalence of cardiometabolic multimorbidity was 14.43% (13.67–15.22); and while being employed, wealthy, and having high educational level were negatively associated with the prevalent cardiometabolic multimorbidity, aging, urban residency, physical inactivity, central and general obesity, positive family history of MI/Stroke, positive family history of diabetes, and history of COVID-19 hospitalization had positive associations (all p-values < 0.05). Moreover, sex-stratified analyses showed similar results. As a limitation, history of MI/stroke was based on self-reported data. In conclusion, 14.43% of Iranian adults experience cardiometabolic multimorbidity with comparable prevalence between men and women, posing a considerable burden on public health and underscoring the necessity for pragmatic and multicomponent preventive strategies in both sexes.

**Supplementary Information:**

The online version contains supplementary material available at 10.1038/s41598-026-52569-6.

## Introduction

According to the Global Burden of Disease (GBD) study, 37.2% of global population were suffering from multimorbidity (defined as presence of at least two non-communicable diseases (NCDs) simultaneously) in 2023 and the corresponding prevalence in the middle east region were estimated as 21.8%^1,2^. Moreover, other study from GBD 2023 revealed that cardiometabolic conditions, including cardiovascular diseases (CVD), hypertension (HTN), and diabetes mellitus remain as the leading NCDs responsible for substantial burden of global mortality; and particularly in the Middle East and North Africa (MENA) region^3^. Importantly, based on their shared and interconnected pathophysiology, clinicians commonly encounter patients with concurrent type 2 diabetes (T2DM), HTN, and CVD, a pattern referred to as cardiometabolic multimorbidity^4–6^, which increases with older age and is associated with more complicated healthcare needs, reduced quality of life, and an increased rate of mortality^5^.

The majority of clinical guidelines and healthcare practitioners tend to prioritize a single disease, which can result in care that is occasionally insufficient and may not consider the overall person’s health. However, it is essential to understand cardiometabolic multimorbidity patterns and their related factors for a more comprehensive understanding of this prevalent pattern^7^. A national study conducted among U.S. adults estimated the prevalence of cardiometabolic multimorbidity at 14.4%, with higher prevalence observed among males, older individuals, and non-Hispanic Black populations^8^. This knowledge can improve clinical decision-making and inform public health planning. Of note, various factors are associated with cardiometabolic multimorbidity, i.e., aging, sex, and broader health determinants such as socioeconomic disadvantage^9^.

Some studies have reported prevalence of multimorbidity across different provinces of Iran, ranging from 36.6% in northern areas to 24.4% in southern areas^10,11^; however, conducting studies among special populations (i.e., older individuals and hospitalized patients), using different definitions of multimorbidity, and absence of a national study have limited our knowledge. On the other hand, given the rapidly rising use of medications with multidimensional cardiometabolic impacts (including but not limited to sodium-glucose cotransporter-2 inhibitors and glucagon-like peptide-1 receptor agonists), there is an important gap in the current knowledge regarding the prevalence of patients with cardiometabolic multimorbidity^12^. Therefore, in the present study using data from national representative samples of the 2021 WHO STEPwise Approach to Non-communicable Disease (NCD) Risk Factor Surveillance (STEPS) survey, we aimed to evaluate the prevalence of cardiometabolic multimorbidity and its association with social determinants of health among a general Iranian population.

## Materials and methods

### Study design

This study used data from the 2021 Iran STEPS Survey, a nationally representative survey designed to evaluate NCDs across 31 provinces of Iran. All methods were carried out in accordance with relevant guidelines and regulations, including the World Health Organization’s STEPS protocol and ethical standards for human research. The study protocol was approved by the Ethics Committee of Shahid Beheshti University of Medical Sciences (Approval ID: IR.SBMU.MSP.REC.1403.486), ensuring compliance with ethical standards for research involving human participants. Informed consent was obtained from all participants or their legal guardians prior to their inclusion in the Iran STEPS Survey 2021, in accordance with the study protocol. Detailed information regarding the Iran STEPS Survey 2021 can be found in the study protocol^13^. This survey was conducted in three major and distinct steps of data collection: questionnaires, physical measurements, and laboratory tests. The first step followed the most recent version (version 3.2) of the WHO STEPS instrument (27,874 individuals aged ≥ 18 completing the first step)^14^. The second step involved measuring participants’ height, weight, blood pressure, and waist circumference (WC) according to the guidelines outlined in the survey protocol (27,745 individuals aged ≥ 18 completing the second step). The third step included laboratory analyses, and as Iran-STEPS protocol, laboratory assessments were performed only among individuals aged ≥ 25 years (18,119 individuals aged ≥ 25 completing the third step).

### Study population

In the current study, from 18,119 individuals aged ≥ 25 years who had laboratory assessments, first we excluded participants aged ≥ 75 years (*N* = 861), resulting in 17,258 eligible participants aged between 25 and 75 years for the analyses. Next, subjects with missing data on covariates including age, sex, area, job, marital status, physical activity, education levels, smoking status, ever alcohol use, weight, height, WC, fruit servings per week, vegetable servings per week, nuts intake, wealth index (WI), body mass index (BMI), family history of diabetes, family history of MI/stroke, education levels, hospitalized for covid-19, systolic blood pressure (SBP), diastolic blood pressure (DBP), fasting blood glucose (FBS) and hemoglobin A1C (HbA1C) (*n* = 805; considering overlap features between numbers) were excluded; leading to a total sample of 16,453 participants (7,264 men and 9,189 women) for the current analyses (Figure S1).

### Clinical and laboratory measurements

Demographic data regarding sex, age, education levels, occupation, marital status, basic insurance, and household assets, along with information on cigarette smoking, physical activity, alcohol use, medication use and medical history, were collected from participants through a standardized questionnaire derived from the STEPS survey. Participants’ physical activity was evaluated utilizing the Global Physical Activity Questionnaire (GPAQ)^15^. The questionnaire includes 16 questions that measure the intensity, duration, and frequency of physical activity. The data collected from the GPAQ were then reported as metabolic equivalents (METs)-minutes per week^16^. Fruit, vegetables, and nuts intake were defined as an individual’s daily consumption.

Weight was recorded in kilograms while wearing minimal clothing utilizing a standard digital scale (Inofit), which was calibrated with a 5 kg reference scale before each use, whenever the device was relocated. Height was measured while standing barefoot using a measuring tape, with a possible error margin of 0.5 cm. WC was taken at the midpoint between the lowest rib and the hip, just above the umbilicus, along a straight line. SBPs and DBPs were recorded three times on the brachial artery, with a three-minute gap between each reading, after 15 min of rest in a seated position, using standard Beurer sphygmomanometers. The ultimate blood pressure value was finally calculated as the average of the second and third measures. Laboratory analyses were carried out at the survey headquarters utilizing an auto-analyzer (Roche-Hitachi Cobas C311, High-Technologies Corporation, Tokyo, Japan), with approval from the reference laboratory. FPG was recorded utilizing commercial kits and the biochemistry auto analyzer (Cobas C311 Hitachi High–Technologies Corporation. Tokyo, Japan) and HbA1c via High-Performance Liquid Chromatography (HPLC), approved by Health Reference. Inter- and intra-assay coefficients of variation were less than 3% for all the biomarkers used in this study^17^.

### Variable definitions

BMI was calculated by dividing weight in kilograms by height in meters squared (kg/m²), and classified as < 25 kg/m², 25–29.9 kg/m², and ≥ 30 kg/m². According to the nationally validated cutoff points for Iranian adults, abdominal obesity was defined as WC ≥ 95 cm^18^. The wealth index (WI), as the socio-economic status, was calculated from household assets and divided into five quintiles spanning from the poorest to the wealthiest. Education levels were categorized by years of schooling: 0, 1–6, 7–11, and ≥ 12 years. Job status was also categorized into four subtypes, including unemployed, employed, unpaid work, and retired. Alcohol consumption was classified as ever or never used, and smoking status was categorized as current, never, or past smoker. Physical activity was measured in total minutes per week, with < 150 min/week considered less active. Family history of MI/Stroke and diabetes was recorded as present or absent, and COVID-19 hospitalization was categorized as yes or no.

### Morbidity definitions

HTN was determined by DBP ≥ 90 mmHg, SBP ≥ 140 mmHg, or antihypertensive medication use. T2DM was diagnosed with HbA1c ≥ 6.5%, or FBS ≥ 126 mg/dL or diabetes medication use. Myocardial infarction (MI)/Stroke history was evaluated based on self-report by asking “Has any healthcare worker told you that you had MI/Stroke?”. Cardiometabolic multimorbidity was defined as having two or more chronic conditions^19^, including HTN, T2DM, and MI/Stroke.

### Statistical analysis

All the analyses had a weighting plan executed in four consecutive steps after data cleaning. The first step involved the application of total non-response weights to adjust for refusals to answer. Adjustments were particularly tailored for different age groups, taking into account the effect of the COVID-19 pandemic. Afterwards, samples from each province were weighted by sex, age, and residence place to attain demographic representativeness. To obtain more details on the weighting strategy, see the study protocol^13^. Descriptive statistics were represented as prevalence estimates, with 95% confidence intervals (CIs), and for continuous variables mean (standard error: SE) was considered. The difference between categorical variables among different categories of morbidities (i.e., 0, 1, or ≥ 2) was assessed by chi-square test, and between continuous variables by one-way ANOVA. Multicollinearity of independent variables was checked via variance inflation factor (VIF) statistics (VIF < 5 shows acceptable collinearity). Stepwise logistic regression for cardiometabolic multimorbidity was done through a stepwise backward elimination process, with an inclusion criterion of 0.2 (to reduce the risk of excluding potentially important covariates), which is more liberal than the conventional 0.05 level. Statistical significance was ascertained utilizing a 95% CI in combination with a p-value of less than 0.05. The interaction between sex and potential covariates in multivariable models was also reported. Considering the number of variables in the final model (19 variables), a Bonferroni correction was employed, resulting in the significancy level of 0.002; and no statistically significant interaction was found. Statistical analysis was performed with Stata 17.

##  Results

A total of 16,453 subjects (men: 45.33%, women: 54.67%), mean (SE) age of 46.90 (0.15) years, were included in the current data analysis. Table 1 displays demographic characteristics of participants according to the number of cardiometabolic morbidities. Compared to the individuals with < 2 morbidities, their counterparts with cardiometabolic multimorbidity were older and had higher levels of BMI, WC, SBP, FBS, and HbA1c values (all P-values < 0.05). Moreover, the prevalence of individuals with urban residency, basic health insurance, low educational level, retirement status, physical activity < 150 min/week, past smoking, consuming < 3 servings of vegetables per week and < 3 servings of nuts per months, family history of MI/stroke, family history of diabetes, and hospitalization for covid-19 was higher among participants with cardiometabolic multimorbidity (all p values < 0.05). Evaluating demographic characteristics among men and women separately showed that women had higher BMI levels than men (28.32 vs. 26.30 kg/m²), whereas the opposite pattern was observed for SBP (125.91 vs. 127.31 mmHg) and DBP (77.92 vs. 79.34 mmHg) (Tables S1 and S2). The VIF values for independent variables were range from 1.4 to 4 less (e.g. both BMI and WC were 2.30) that shows low level of collinearity.

**Table 1:** Demographic characteristics of participants according to the number of cardiometabolic morbidities; Iran STEPS-2021.

Socio-demographic factors		Number of cardiometabolic morbidities			*p*-value
	Total (*n* = 16453)	0 (*n* = 9135)	1 (*n* = 4699)	≥ 2 (*n* = 2619)	
Categorical variables, prevalence% (95% CI)					
Age groups, years					
25–35	20.91 (20.0–21.85.0.85)	87.38 (85.35–89.17)	11.62 (9.88–13.63)	1.00 (0.63–1.58)	< 0.01
35–45	25.86 (24.9–26.84.9.84)	75.27 (73.30–77.15.30.15)	20.90 (19.20–22.71.20.71)	3.83 (2.91–5.03)	
45–55	22.35 (21.48–23.24)	52.77 (50.59–54.94)	33.76 (31.76–35.81)	13.48 (12.01–15.09)	
55–65	19.37 (18.52–20.26)	30.81 (28.58–33.14)	39.91 (37.48–42.40)	29.27 (27.07–31.58)	
65–75	11.51 (10.82–12.24)	21.86 (19.03–25.00.03.00)	38.67 (35.59–41.85)	39.46 (36.36–42.65)	
Sex					
Women	54.67 (53.57–55.77)	57.76 (56.31–59.20)	27.28 (26.01–28.58)	14.96 (13.93–16.04)	0.32
Men	45.33 (44.23–46.43)	58.31 (56.66–59.95)	27.90 (26.42–29.44)	13.78 (12.69–14.96)	
Area					
Urban	75.43 (74.63–76.21)	57.48 (56.13–58.81)	27.36 (26.17–28.59)	15.16 (14.22–16.15)	< 0.01
Rural	24.57 (23.79–25.37)	59.65 (58.05–61.24)	28.18 (26.75–29.66)	12.16 (11.13–13.27)	
Basic health insurance					
Yes	90.38 (89.65–91.06)	56.75 (55.61–57.88)	28.06 (27.03–29.10)	15.19 (14.39–16.04)	< 0.01
No	9.623 (8.94–10.35)	69.87 (66.35–73.17)	22.92 (20.01–26.12)	7.20 (5.47–9.44)	
Job					< 0.01
Unemployed	4.09 (3.73–4.50)	59.72 (55.11–64.17)	25.68 (21.91–29.86)	14.59 (11.88–17.80)	
Employed	40.38 (39.29–41.48)	68.14 (66.46–69.78)	24.16 (22.66–25.73)	7.69 (6.83–8.66)	
Unpaid works	45.71 (44.63–46.81)	54.96 (53.41–56.51)	29.30 (27.90–30.75.90.75)	15.73 (14.65–16.88)	
Retired	9.81 (9.13–10.53)	29.81 (26.57–33.27)	34.23 (30.77–37.86)	35.96 (32.30–39.79.30.79)	
Marital status					
Single	8.54 (7.91–9.22)	79.43 (75.80–82.63.80.63)	17.07 (14.26–20.31)	3.50 (1.98–6.12)	< 0.01
Married	83.31 (82.44–84.14)	57.68 (56.50–58.86.50.86)	27.80 (26.94–29.07)	14.32 (13.50–15.18.50.18)	
Divorced/widow	8.15 (7.56–8.78)	38.94 (35.22–42.79)	34.14 (30.43–38.05)	26.93 (23.71–30.41)	
Education levels, years					
0	12.72 (12.11–13.35)	32.71 (30.33–35.18)	37.89 (35.53–40.31)	29.40 (27.13–31.78)	< 0.01
1 to < 7	25.4 (24.53–26.29)	47.77 (45.87–49.67)	33.33 (31.56–35.15)	18.91 (17.41–20.50)	
≥ 7 to < 12	20.02 (19.18–20.89)	63.20 (60.86–65.48)	26.66 (24.61–28.82)	10.14 (8.71–11.77)	
≥ 12	41.86 (40.74–43.00.74.00)	69.44 (67.57–71.25)	21.36 (19.75–23.06)	9.21 (8.09–10.45)	
physical activity, min/week					
< 150	46.06(45.00–47.16.00.16)	55.4(53.76–57.03)	27.41(26.00–28.88.00.88)	17.19(16.00–18.46.00.46)	
≥ 150	53.94(52.84–55.04)	60.24(58.78–61.68)	27.46(26.15–28.81)	12.3(11.35–13.31)	
Smoking status					
Never	79.69 (78.77–80.58)	57.65 (56.44–58.85)	27.91 (26.84–29.01)	14.44 (13.60–15.33.60.33)	< 0.01
Past smoker	5.61 (5.14–6.11)	50.54 (46.14–54.94)	30.10 (25.95–34.60)	19.36 (16.25–22.91)	
Current smoker	14.71 (13.91–15.54)	62.85 (59.83–65.78)	24.71 (22.12–27.50)	12.44 (10.53–14.64)	
Ever alcohol use					0.09
Yes	7.12 (6.52–7.78)	63.15 (58.36–67.71)	24.77 (20.63–29.43)	12.08 (9.28–15.58)	
No	92.88 (92.22–93.48)	57.62 (56.50–58.73.50.73)	27.78 (26.79–28.79)	14.60 (13.83–15.42)	
BMI categories, kg/m^2^					
< 25	32.72 (31.70–33.74.70.74)	72.19 (70.47–73.83)	19.76 (18.40–21.20)	8.05 (6.94–9.32)	< 0.01
≥ 25 to < 30	40.29 (39.20–41.39.20.39)	57.46 (55.70–59.21.70.21)	28.90 (27.30–30.55.30.55)	13.64 (12.55–14.80)	
≥ 30	27.00 (26.04–27.97)	41.66 (39.66–43.68)	35.02 (33.04–37.05)	23.33 (21.61–25.14)	
WC, cm					
< 95	49.85 (48.75–50.95)	71.85 (70.40–73.25.40.25)	21.02 (19.79–22.31)	7.13 (6.29–8.08)	< 0.01
≥ 95	50.15 (49.05–51.25)	44.26 (42.74–45.79)	34.06 (32.61–35.54)	21.67 (20.47–22.93)	
Fruit serving, per week					
< 6	36.55 (35.51–37.60)	57.24 (55.49–58.98)	28.86 (27.26–30.52)	13.89 (12.73–15.15)	0.22
≥ 6 to < 15	41.89 (40.79–43.00.79.00)	58.11 (56.36–59.85)	26.72 (25.20–28.29.20.29)	15.17 (13.96–16.47)	
≥ 15	21.56 (20.70–22.45.70.45)	59.12 (56.86–61.34)	27.00 (25.04–29.05)	13.88 (12.31–15.62)	
Vegetables serving, per week					
< 3	32.90 (31.88–33.95)	55.76 (53.83–57.68)	28.80 (27.05–30.62)	15.43 (14.05–16.93)	0.04
≥ 3 to < 9	34.65 (33.61–35.71)	58.86 (57.01–60.69)	26.67 (25.09–28.32)	14.47 (13.18–15.86)	
≥ 9	32.45 (31.43–33.48)	59.39 (57.52–61.23)	27.25 (25.60–28.97.60.97)	13.36 (12.19–14.62)	
Nuts intake					
< 3 times per month	61.80 (60.72–62.80)	54.12 (52.72–55.51)	29.00 (27.74–30.28)	16.89 (15.83–18.00.83.00)	< 0.01
1–6 times a week	32.60 (31.54–33.63)	64.60 (62.74–66.42)	25.26 (23.61–26.99)	10.14 (9.15–11.23)	
> 1 times a day	5.63 (5.18–6.11)	62.63 (58.59–66.51)	25.17 (21.83–28.83)	12.20 (9.92–14.91)	
Wealth index, quintile					
1 st (lowest)	18.33 (17.54–19.15)	56.78 (54.46–59.07)	27.69 (25.72–29.75)	15.53 (14.04–17.14)	0.05
2nd	19.97 (19.09–20.89)	56.18 (53.64–58.70)	27.46 (25.25–29.78)	16.36 (14.51–18.39)	
3rd	19.30 (18.53–20.10)	57.36 (55.20–59.49.20.49)	28.47 (26.60–30.43.60.43)	14.17 (12.72–15.76)	
4th	20.63 (19.79–21.49)	59.25 (56.99–61.47)	26.62 (24.68–28.66)	14.13 (12.53–15.89)	
5th	21.77 (20.76–22.81)	60.14 (57.34–62.87)	27.63 (25.12–30.29)	12.23 (10.51–14.19)	
Family history of MI/stroke					
Yes	13.29 (12.54–14.07)	49.04 (45.96–52.13)	31.11 (28.41–33.96)	19.85 (17.37–22.58)	< 0.01
No	86.71 (85.93–87.46)	59.39 (58.23–60.54)	27.02 (25.98–28.08)	13.59 (12.82–14.41)	
Family history of diabetes					
Yes	32.11 (31.11–33.12)	48.37 (46.52–50.23)	29.88 (28.18–31.64)	21.74 (20.20–23.37.20.37)	< 0.01
No	67.89 (66.88–68.89)	62.57 (61.25–63.87)	26.47 (25.29–27.67)	10.96 (10.16–11.83)	
Hospitalized for Covid-19					
Yes	1.05 (0.863–1.27)	42.08 (32.95–51.78)	23.81 (15.96–33.96)	34.11 (26.00–43.27.00.27)	< 0.01
No	98.95 (98.73–99.14)	58.18 (57.09–59.27)	27.60 (26.63–28.60)	14.22 (13.46–15.01)	
Continuous variables, mean (SE)					
Age, years	46.90 (0.15)	41.56 (0.17)	52.00 (0.26)	58.91 (0.29)	< 0.01
BMI, kg/m^2^	27.40 (0.05)	26.34 (0.06)	28.53 (0.10)	29.55 (0.14)	< 0.01
WC, cm	94.47 (0.15)	91.19 (0.18)	97.68 (0.27)	101.62 (0.42)	< 0.01
SBP, mmHg	126.54 (0.19)	117.00 (0.14)	137.91 (0.38)	143.32 (0.55)	< 0.01
DBP, mmHg	78.57 (0.13)	74.03 (0.11)	85.01 (0.27)	84.54 (0.32)	< 0.01
FBS, mg/dL	104.82 (0.36)	93.92 (0.13)	109.26 (0.82)	143.67 (1.53)	< 0.01
HbA1c, %	6.00 (0.01)	5.55 (0.006)	6.17 (0.03)	7.50 (0.05)	< 0.01

BMI: body mass index, WC: waist circumference, SBP: systolic blood pressure, DBP: diastolic blood pressure, FBS: fasting blood glucose, HbA1c: hemoglobin A1c.

P values < 0.05 considered statistically significant

**Fig. 1:**
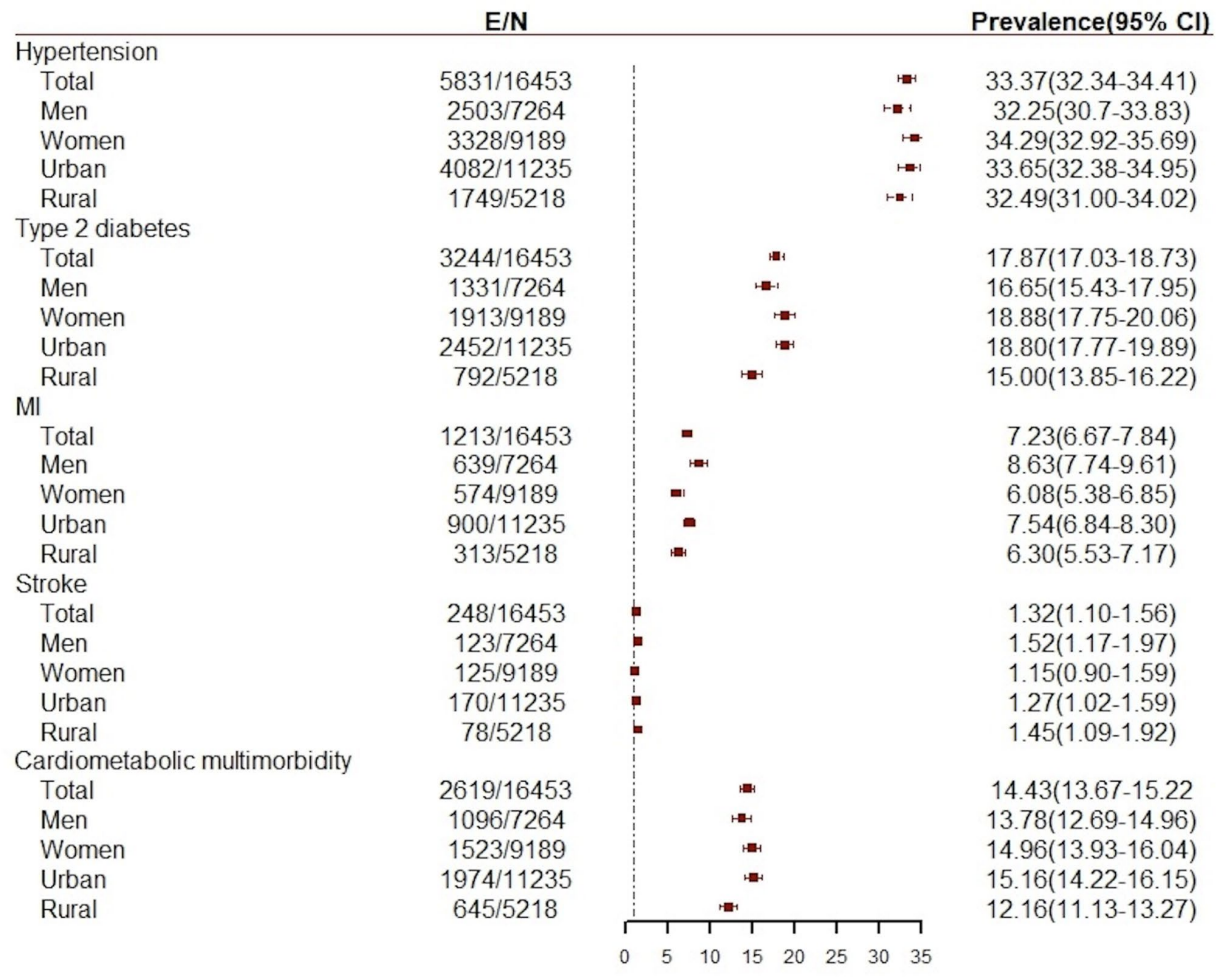
Prevalences (95% confidence intervals) of cardiometabolic multimorbidity and each morbidity irrespective of the presence of others, stratified by sex and residency area; Iran-STEPS 2021.

Table 2 presents the prevalence of individuals with specific morbidity combinations. Accordingly, 58.01% (56.92–59.09) had no morbidity, 27.56% (26.59–28.55) had only one morbidity (HTN: 19.78% (18.93–20.67) or T2DM: 6.02% (5.51–6.57) or MI/Stroke: 1.76% (1.50–2.07)), 11.73% (11.07–12.45) had only two morbidities, and 2.68% (2.31–3.11) had all three (HTN+T2DM + MI/Stroke). For women, 57.76% (56.31–59.20) had no morbidity, 27.28% had only one, 12.32% had only two, and 2.64% had all three morbidities; the corresponding values among men were 58.31%, 27.91%, 11.04%, and 2.74%, respectively. In total, 14.43% (13.67–15.22) had cardiometabolic multimorbidity, with no statistically significant difference between men and women (men: 13.78% (12.69–14.96); women: 14.96 (13.93–16.04); P value > 0.05) (Fig. 1).

**Table 2:** Prevalence of cardiometabolic multimorbidity and distribution of morbidities among women, men, and in the total population.

Number of morbidities	Morbidities	Total population		Women		Men	
		N*	Prevalence (95% CI)	N*	Prevalence (95% CI)	N*	Prevalence (95% CI)
Without cardiometabolic components	-	9135	58.01 (56.92–59.09)	5091	57.76 (56.31–59.20)	4044	58.31 (56.66–59.95)
Only 1 component	HTN	3356	19.78 (18.93–20.67)	1864	19.87 (18.76–21.03)	1492	19.68 (18.36–21.07)
	T2DM	1051	6.02 (5.51–6.57)	585	6.04 (5.39–6.77)	466	5.99 (5.22–6.85)
	MI/Stroke	292	1.76 (1.50–2.07)	126	1.37 (1.05–1.78)	166	2.24 (1.83–2.73)
Only 2 components	HTN+T2DM	1569	8.32 (7.75–8.94)	1003	9.67 (8.83–10.57)	566	6.70 (5.95–7.54)
	HTN + MI/Stroke	426	2.57 (2.27–2.92)	195	2.12 (1.76–2.56)	231	3.12 (2.64–3.69)
	T2DM + MI/Stroke	144	0.84 (0.66–1.07)	59	0.53 (0.39–0.73)	85	1.22 (0.88–1.69)
All 3 components	HTN+T2DM + MI/Stroke	480	2.68 (2.31–3.11)	266	2.64 (2.16–3.21)	214	2.74 (2.20–3.41)

Number of individuals only with the disease(s) in the corresponding row.

HTN, hypertension, T2DM, type 2 diabetes, MI, myocardial infarction; N: number, CI, confidence interval.

Table 3. presented the stepwise logistic regression models to identify the association between socio-demographic parameters and prevalent cardiometabolic multimorbidity. The findings of total population demonstrated that higher age was consistently associated with higher odds of cardiometabolic multimorbidity. Moreover, urban residency (urban vs. rural: 1.32 (1.17–1.49)), higher educational level (≥ 12 vs. 0 years: 0.67 (0.56–0.80)), being employed (employed vs. unemployed: 0.74 (0.57–0.96)), family history of MI/Stroke (with vs. without: 1.42 (1.24–1.62)), family history of diabetes (with vs. without: 2.44 (2.20–2.69), and history of Covid-19 hospitalization (with vs. without: 2.02 (1.43–2.85)) were significantly associated with prevalent cardiometabolic multimorbidity. With regards to the wealth status, participants in the fourth and fifth quintiles showed a significantly lower odds of having cardiometabolic multimorbidity with ORs of 0.82 (0.70–0.96) and 0.75 (0.63–0.90), respectively. Moreover, compared to the BMI < 25 kg/m^2^, being overweight (BMI 25–30 kg/m^2^: 1.51 (1.31–1.75)), general obesity (BMI ≥ 30 kg/m^2^: 2.13 (1.80–2.51)); as well as the central obesity (WC ≥ 95 cm vs. <95 cm: 1.81 (1.59–2.06)), and low physical activity (< 150 vs. ≥150 min/week: 1.20 (1.02–1.42)) were significantly associated with higher odds of having cardiometabolic multimorbidity. Similar patterns of associations were observed in both sexes, without any significant interaction between men and women.

**Table 3.** Stepwise logistic regression for the association between socio-demographic factors and cardiometabolic multimorbidity; Iran STEPS-2021

**Socio-demographic factors**	**Total population**			**Female**			**Male**			p for interaction*
	**E/N**	**OR (95% CI)**	**p-value**	**E/N**	**OR (95% CI)**	**p-value**	**E/N**	**OR (95% CI)**	**p-value**	
Age groups, years										
25-35	32/3222	Reference		14/1829	Reference		18/1394	Reference		0.18
35-45	152/4126	2.85 (1.93-4.20)	<0.01	91/2375	3.72 (2.11-6.59)	<0.01	61/1751	2.30 (1.34-3.93)	<0.01	
45-55	530/3829	10.15 (7.00-14.70)	<0.01	329/2180	13.89 (8.03-24.02)	<0.01	201/1649	7.82 (4.76-12.86)	<0.01	
44-65	1035/3280	27.01 (18.62-39.18)	<0.01	579/1753	36.50 (21.10-63.12)	<0.01	456/1527	21.58 (13.13-35.46)	<0.01	
65-75	870/1996	44.08 (30.06-64.63)	<0.01	510/1053	71.71 (41.07-125.21)	<0.01	360/943	30.49 (18.19-51.12)	<0.01	
Area, urban (ref: rural)	1947/11235	1.32 (1.17-1.49)	<0.01	1116/6280	1.34 (1.15–1.56)	<0.01	858/4955	1.32 (1.10-1.59)	<0.01	0.65
Job										
Unemployed	122/736	Reference		-	-	-	109/583	Reference		0.68
Employed	536/6231	0.74 (0.57-0.96)	0.02	-	-	-	472/5218	0.59 (0.45-0.77)	<0.01	
Unpaid works	1387/7908	0.81 (0.63-1.03)	0.08	-	-	-	15/134	0.63 (0.32-1.23)	0.17	
Retired	574/1578	1.25 (0.96-1.63)	0.10	-	-	-	500/1329	1.09 (0.82-1.46)	0.54	
Marital status										
Single	42/1282	Reference		-	-	-	-	-	-	0.02
Married	2165/13835	1.13 (0.79-1.59)	0.50	-	-	-	-	-	-	
Divorced/widow	412/1333	1.38 (0.95-2.01)	0.09	-	-	-	-	-	-	
Education levels, years										
0	780/2592	Reference		623/1895	Reference		157/697	Reference		0.54
1 to < 7	926/4662	0.87 (0.76-0.99)	0.04	543/2826	0.81 (0.69–0.95)	0.01	382/1836	1.10 (0.86-1.41)	0.44	
≥ 7 to < 12	339/3180	0.72 (0.60-0.86)	<0.01	163/1511	0.70 (0.55–0.89)	<0.01	176/1669	0.83 (0.62-1.11)	0.20	
≥ 12	574/6019	0.67 (0.56-0.80)	<0.01	194/2957	0.57 (0.45–0.72)	<0.01	380/3062	0.83 (0.64-1.09)	0.19	
Physical activity, <150 min/week (ref: ≥ 150 min/week)	2387/14084	1.20 (1.02-1.42)	0.03	**-**	-	-	-	-	-	0.57
BMI categories, kg/m^2^										
<25	442/5447	Reference		202/2482	Reference		240/2965	Reference		0.06
≥25 to <30	1055/6477	1.51 (1.31-1.75)	<0.01	556/3532	1.48 (1.20–1.82)	<0.01	499/2945	1.50 (1.21-1.85)	<0.01	
≥30	1122/4529	2.13 (1.80-2.51)	<0.01	762/3175	1.95 (1.55–2.44)	<0.01	357/1354	2.54 (1.98-3.25)	<0.01	
WC, ≥95 cm (ref: <95 cm)	2015/8429	1.81 (1.59-2.06)	<0.01	1176/4727	1.67 (1.40--1.98)	<0.01	839/3702	1.94 (1.59-2.38)	<0.01	0.23
Wealth index, quintile										
1st (Lowest)	591/3352	Reference		406/2040	Reference		185/1312	-	-	0.18
2nd	576/3163	0.99 (0.85-1.15)	0.91	375/1889	1.02 (0.84–1.23)	0.85	201/1274	-	-	
3rd	526/3559	0.90 (0.77-1.05)	0.18	293/1899	0.90 (0.74–1.10)	0.31	233/1660	-	-	
4th	509/3421	0.82 (0.70-0.96)	0.02	259/1801	0.78 (0.63–0.96)	0.02	250/1620	-	-	
5th	417/2958	0.75 (0.63-0.90)	<0.01	190/1560	0.72 (0.57–0.91)	<0.01	227/1398	-	-	
Family history MI/STROKE, yes (ref: no)	441/2115	1.42 (1.24-1.62)	<0.01	267/1303	1.28 (1.08–1.52)	<0.01	174/812	1.69 (1.36-2.09)	<0.01	0.10
Family history diabetes, yes (ref: no)	1279/5392	2.44 (2.20-2.69)	<0.01	765/3185	2.44 (2.14--2.78)	<0.01	514/2207	2.50 (2.15-2.92)	<0.01	0.18
Hospitalized for Covid-19, yes (ref: no)	77/194	2.02 (1.43-2.85)	<0.01	46/111	1.89 (1.20–3.00)	<0.01	31/83	2.17 (1.27-3.68)	<0.01	0.32

OR: odds ratio; CI: confidence interval. BMI: body mass index; WC: waist circumference; ref: reference.P values < 0.05 considered statistically significantVariables with p values > 0.2 in the multivariable model did not show in the table, including sex, smoking status, alcohol use, fruit servings per week, vegetable servings per week, nuts intake, andbasic health insurance*Bonferroni correction was employed, resulting in the significancy level of 0.002

## Discussion

This national cross-sectional study revealed that approximately 14.4% of Iranian adults had cardiometabolic multimorbidity in 2021, defined as having at least two of HTN, T2DM, and/or MI/stroke. Our results indicated hypertension as the most dominant component (33.37% (32.34–34.41)), as reflected in both individual and combined disease prevalence. Different socio-demographic factors such as ageing, familial predisposition to cardiovascular disease and diabetes, prior COVID-19 hospitalization, urban residency, sedentary behavior, and general/central obesity were associated with prevalent cardiometabolic multimorbidity. Conversely, higher socioeconomic status, advanced education, and being employed demonstrated negative associations.

The findings of this study on the cardiometabolic multimorbidity prevalence in Iran, highlight the importance of considering socio-demographic factors, including aging, central and general obesity, low physical activity, and FH-DM and FH-CVD when evaluating individuals with chronic diseases, which aligns with previous research^20–22^. In other words, our results suggest that cardiometabolic multimorbidity is more prevalent among individuals with poorer socio-demographic status, and physicians have to account for this issue when encounter individuals with poor socio-demographic characteristics. However, the temporal relationship between these associations needs to be assessed through longitudinal studies.

Regarding sex specific prevalence of cardiometabolic multimorbidity, our results demonstrated comparable prevalences between men and women (near 14% and 15%, respectively). Our complementary analysis evaluating demographic characteristics among men and women separately showed that women had higher levels of BMI compared with men (28.32 kg/m^2^ vs. 26.30 kg/m^2^). Similarly, recent results from GBD 2021 have revealed that in 2021, the prevalence of adult females with overweight and obesity was higher than adult males (1·11 billion (1·10 − 1·12) vs. 1·00 billion (0·989-1·01))^23^. Of note, given the substantial role of general obesity on the development of cardiometabolic multimorbidity^24^, existing sex disparities in demographic characteristics underscores the importance of providing sex-specific information. Additionally, another study from GBD 2021 study has shown that while women were suffering from higher morbidity-driven conditions, men had higher burden of mortality-driven conditions in the world^25^; indicating meaningful differences between men and women. Great burden of environmental, psychosocial and behavioral stressors, poorer risk profile, and a higher burden of cardiovascular disease (as the leading cause of death) among men compared with women in the world, as well as Iran, are some other important issues that justify our sex-stratified analyses, and should be considered when interpreting our results^26–29^. Therefore, future longitudinal researches are required to evaluate cardiometabolic multimorbidity onset and development process among men and women separately.

In this study, urbanization was associated with cardiometabolic multimorbidity, independent of physical activity, with odds ratio (95% CI) of 1.32 (1.17–1.49) in the stepwise logistic regression analysis. This finding is consistent with the previous studies^30^. The relationship between urban living and cardiometabolic multimorbidity is likely influenced by several factors commonly found in urban environments, such as diet, air pollution, stress, and socioeconomic conditions^31^. Among Iranian population residents in Tehran, we showed an unfavorable impact of air pollution on the development of hypertension, dysglycemia, and CVDs; which are components of cardiometabolic multimorbidity^32,33^.

Individuals in the highest wealth quintiles were significantly less likely to have cardiometabolic multimorbidity. Similar to our results, a study across 41 countries found that as wealth increases, the prevalence of multimorbidity decreases^34^. In addition, a study by Safari Faramani et al.^35^ demonstrated lower multimorbidity rates among individuals with higher socioeconomic status. Economic inequality is associated with disparities in education, healthcare services, nutrition, and access to clean water, all of which have a substantial impact on health outcomes^36^. Similar results were observed in women, which can be attributed to psychological safety. A study involving 96,169 individuals from 114 countries found that women experience higher financial anxiety compared to men, especially in less-developed countries^37^. Nevertheless, Dolui et al.^38^ and Hossain et al.^39^ observed that individuals from higher socioeconomic groups had a greater likelihood of experiencing multimorbidity. This pattern may be explained by higher rates of disease detection and greater survey participation among individuals from higher socioeconomic groups. Additionally, cultural differences between our study population and those in previous studies may also account for the observed discrepancies. Economic studies exploring the relationship between wealth and health frequently report inconsistent results^40^.

In line with our findings, Tan et al.^41^, Frølich et al.^42^, and Chlabicz et al.^43^ demonstrated that the prevalence of multimorbidity was negatively associated with educational attainment. North et al.^44^ in a Mendelian randomization study identified education as a causal risk factor for multimorbidity. They demonstrated that an increase of one standard deviation (equivalent to 5.1 years) in genetically-predicted years of education reduces the risk of multimorbidity by 9.0% (95% CI: 6.5–11.4%). Moreover, the authors found that general adiposity and smoking account for about 32% of the educational inequality in multimorbidity. In the current study, we also found that higher education was associated with lower prevalence of cardiometabolic multimorbidity in the whole population independent of both general and central adiposity; this issue that mainly found among women. Alimohammadian et al.^45^ represented higher levels of education, in contrast to illiteracy, to be inversely associated with multimorbidity, especially in women (p-value < 0.001). An increase in education is associated with better health-related knowledge, which can influence lifestyle behaviors and further decrease the risk of multimorbidity^46^.

Our findings align with the meta-analysis by Goel et al.^47^, which revealed that the pooled odds of multimorbidity were lower in employed individuals compared to those who were not employed. This aligns with a systematic review of studies carried out in the WHO Eastern Mediterranean countries^48^. Unemployed individuals may experience financial challenges, as well as physical and mental health issues^49^. It should also be noted that people with multiple health conditions are more likely to leave the workforce, leaving behind those who are healthier^50^. For example, the studies conducted by Pedron et al.^51^ and Leal et al.^52^ have found a link between long-term health conditions, including diabetes and cardiovascular diseases, and unemployment, respectively.

Our analysis also found that Individuals who have been hospitalized for COVID-19 had higher odds of cardiometabolic multimorbidity. They represent a group that is generally in a more unstable health condition, despite making up only 1% of the total study sample. However, given the crass-sectional nature of our study, we could not evaluate the temporal relationship between COVID-19 hospitalization and prevalent cardiometabolic multimorbidity. Based on a study by Carmona-Pírez et al.^53^ on 48,415 COVID-19 patients, 80% had multimorbidity. They found that multimorbidity patterns, primarily involving cardiometabolic diseases, were most consistently linked to the severity of infection. Furthermore, according to a study by Chudasamathe et al.^54^ on the UK Biobank participants, the likelihood of experiencing a severe SARS-CoV-2 infection rose with the number of pre-existing conditions included in the multimorbidity index. Additionally, Catalano et al.^55^ reported multimorbidity as a contributing factor to a more severe outcome in COVID-19. Of note, the association between COVID-19 severity and cardiometabolic multimorbidity appears to be bidirectional; not only are individuals with pre-existing cardiometabolic multimorbidity more prone to severe COVID-19^54,56^, but a higher incidence of cardiometabolic morbidities following severe COVID-19 infection has also been reported^57^. Therefore, longitudinal studies are required to extend our understanding in this regard.

A major strength of this study is the availability of the large representative national sampling data from the STEPS Survey 2021 of Iran, which would generalize the findings in the adult population of Iran. The comprehensive data collection had included demography, clinical, laboratory, and lifestyle variables to enable a robust analysis of associations between several factors and cardiometabolic multimorbidity. Besides, standardized methods for physical and laboratory measurements have increased the reliability of the results.

Nevertheless, some limitations should be taken into account. In general, the cross-sectional design precludes any conclusions about the temporal difference between men and women in the process of developing cardiometabolic multimorbidity. Some of the self-report variables, namely physical activity, history of myocardial infarction/stroke, smoking, alcohol use, COVID-19 hospitalization are subject to recall or social desirability bias; which can cause underestimation. In addition, as healthier individuals have higher probability of being hired and able to work, “healthy worker effect” should be considered regarding the association between employment status and cardiometabolic multimorbidity. Furthermore, this study has been conducted among participants aged between 25 and 75 years, and obtained results would not be extrapolatable to younger and older individuals. Finally, cultural differences, as well as health-seeking behavior, could also affect how detection and reporting of chronic diseases are done.

## Conclusion

In summary, this nationally representative study highlights that 14.43% of Iranian adults experience cardiometabolic multimorbidity with comparable prevalence between men and women, and hypertension emerged as the most prevalent morbidity. Key socio-demographic factors that were positively associated with cardiometabolic multimorbidity included older ages, urban residency, illiteracy, physical inactivity, both general and central obesity, positive family history of CVD and diabetes, as well as lower levels of education and wealth, reflecting the interplay between social engagement, financial security, and cardiometabolic health status.

## Supplementary Information

Below is the link to the electronic supplementary material.

Supplementary Material 1

## Data Availability

Data from this study is available upon a reasonable request from the corresponding authors.
