# Supplementary material for: Cardiometabolic multimorbidity prevalence and its socio-demographic determinants among Iranian adults: insights from the nationwide STEPS 2021
Source: Sci Rep. 2026 Jun 1;16:25024. doi: 10.1038/s41598-026-52569-6 (PMC13462752; doi:10.1038/s41598-026-52569-6)
Supplement: Supplementary file 1 [file 41598_2026_52569_MOESM1_ESM.docx]

**Supplementary Materials**

**Cardiometabolic multimorbidity prevalence and its socio-demographic determinants among Iranian adults: insights from the nationwide STEPS 2021**

### Mohammad Rahmanian, Danial Molavizadeh, Samaneh Akbarpour, Niloofar Deravi, Samaneh Asgari, Farzad Hadaegh

**Table of content**

**Table S1:** Demographic characteristics of women according to the number of cardiometabolic morbidities; Iran STEPS 2021……………………………………………………………………………………………………………………...…….…... page 2

**Table S2:** Demographic characteristics of men according to the number of cardiometabolic morbidities; Iran STEPS-2021……………………………………………………………………………….………………………………………………... page 5

**Figure S1:** Study flow-chart………………………………………………………………………………………………………...page 8

| Table S1: Demographic characteristics of women according to the number of cardiometabolic morbidities; Iran STEPS-2021 | | | | | |
| --- | --- | --- | --- | --- | --- |
| Socio-demographic factors |  | **Number of cardiometabolic morbidities** | | | **p-value** |
|  | **Total (n=9189)** | **0 (n=5091)** | **1 (n=2575)** | **≥2 (n=1523)** |  |
| Age groups, years | | | | | |
| - 25-35 | 20.60 (19.40 - 21.86) | 90.70 (88.42-92.56) | 8.32 (6.54-10.54) | 0.98 (0.53-1.80) | <0.01 |
| - 35-45 | 26.63 (25.35 - 27.95) | 76.42 (73.79-78.87) | 19.47 (17.32-21.81) | 4.11 (2.81-5.96) |  |
| - 45-55 | 22.83 (21.67 - 24.03) | 50.59 (47.70-53.47) | 35.59 (32.93-38.34) | 13.83 (12.00-15.88) |  |
| - 55-65 | 18.87 (17.74 - 20.06) | 28.20 (25.17-31.43) | 41.24 (37.93-44.64) | 30.56 (27.52-33.78) |  |
| - 65-75 | 11.07 (10.18 - 12.02) | 16.77 (13.44-20.74) | 40.41 (36.21-44.76) | 42.82 (38.53-47.22) |  |
| Area | | | | | |
| - Urban | 75.86 (74.8 - 76.89) | 57.85 (56.06-59.61) | 26.77 (25.23-28.37) | 15.38 (14.12-16.74) | 0.11 |
| - Rural | 24.14 (23.11 - 25.2) | 57.50 (55.31-59.66) | 28.88 (26.93-30.91) | 13.62 (12.21-15.18) |  |
| Basic health insurance | | | | | |
| - Yes | 91.23 (90.23 - 92.14) | 56.59 (55.08-58.08) | 27.86 (26.53-29.22) | 15.56 (14.48-16.7) | <0.01 |
| - No | 8.76 (7.86 - 9.77) | 70.01 (64.81-74.74) | 21.28 (17.42-25.72) | 8.71 (5.87-12.74) |  |
| Job | | | | | <0.01 |
| - Unemployed | 1.70 (1.39 - 2.08) | 72.65 (62.43-80.93) | 19.53 (12.17-29.83) | 7.83 (4.26-13.94) |  |
| - Employed | 13.10 (12.04 - 14.24) | 80.35 (76.90-83.40) | 14.48 (11.92-17.47) | 5.17 (3.57-7.42) |  |
| - Unpaid works | 82.04 (80.77 - 83.24) | 54.86 (53.28-56.42) | 29.27 (27.85-30.72) | 15.88 (14.77-17.04) |  |
| - Retired | 3.16 (2.61 - 3.82) | 31.51 (23.82-40.37) | 32.95 (25.39-41.52) | 35.53 (25.79-46.64) |  |
| Marital status | | | | | |
| - Single | 7.71 (6.94 - 8.54) | 79.25 (73.69-83.89) | 16.78 (12.84-21.63) | 3.97 (1.69-9.03) | <0.01 |
| - Married | 79.11 (77.90 - 80.28) | 58.94 (57.31-60.55) | 27.4 (25.98-28.87) | 13.66 (12.57-14.83) |  |
| - Divorced/widow | 13.18 (12.24 - 14.18) | 38.14 (34.34-42.1) | 32.68 (29.21-36.36) | 29.18 (25.71-32.9) |  |
| Education levels, years | | | | | |
| - 0 | 17.08 (16.14 - 18.06) | 30.21 (27.5-33.08) | 38.63 (35.85-41.5) | 31.15 (28.48-33.95) | <0.01 |
| - 1 to <7 | 27.51 (26.31 - 28.75) | 47.23 (44.78-49.7) | 34.96 (32.59-37.41) | 17.81 (15.97-19.8) |  |
| - ≥7 to <12 | 17.38 (16.30 - 18.52) | 63.71 (60.23-67.05) | 25.16 (22.32-28.24) | 11.13 (8.843-13.92) |  |
| - ≥12 | 38.03 (36.54 - 39.55) | 75.03 (72.47-77.43) | 17.59 (15.56-19.83) | 7.37 (5.89-9.20) |  |
| physical activity | | | | | |
| - <1500 min/week | 94.09 (93.41 - 94.70) | 57.58 (56.08-59.07) | 27.16 (25.85-28.51) | 15.25 (14.18-16.39) | 0.04 |
| - ≥1500 min/week | 5.91 (5.30 - 6.59) | 60.62 (55.2-65.79) | 29.14 (24.48-34.27) | 10.24 (7.64-13.61) |  |

| Table S1-continued: Demographic characteristics of women according to the number of cardiometabolic morbidities; Iran STEPS-2021 | | | | | |
| --- | --- | --- | --- | --- | --- |
| Socio-demographic factors |  | **Number of cardiometabolic morbidities** | | | **p-value** |
|  | **Total (n=9189)** | **0 (n=5091)** | **1 (n=2575)** | **≥2 (n=1523)** |  |
| Smoking status | | | | | |
| - Never | 92.69 (91.95 - 93.37) | 57.62 (56.1-59.13) | 27.57 (26.25-28.94) | 14.8 (13.74-15.94) | 0.10 |
| - Past smoker | 2.65 (2.26 - 3.10) | 53.97 (46.04-61.71) | 25.01 (18.49-32.9) | 21.02 (15.68-27.58) |  |
| - Current smoker | 4.66 (4.11 - 5.28) | 62.74 (56.6-68.49) | 22.7 (18.28-27.82) | 14.56 (10.57-19.73) |  |
| Ever alcohol use | | | | | |
| - Yes | 98.80 (98.49 - 99.05) | 76.59 (65.99-84.65) | 14.22 (8.35-23.18) | 9.19 (4.33-18.45) | <0.01 |
| - No | 1.20 (0.95 - 1.51) | 57.53 (56.07-58.98) | 27.44 (26.16-28.75) | 15.03 (13.99-16.12) |  |
| BMI categories, kg/m2 | | | | | |
| - <25 | 26.69 (25.42 - 28.01) | 74.23 (71.68-76.63) | 18.05 (16.12-20.17) | 7.71 (6.11-9.69) | <0.01 |
| - ≥25 to <30 | 39.32 (37.87 - 40.78) | 59.80 (57.42-62.12) | 27.39 (25.3-29.59) | 12.81 (11.41-14.36) |  |
| - ≥30 | 34.00 (32.64 - 35.37) | 42.48 (40.12-44.87) | 34.39 (32.14-36.71) | 23.13 (21.08-25.31) |  |
| WC, cm | | | | | |
| - <95 | 50.77 (49.30 - 52.23) | 73.01 (71.05-74.88) | 19.87 (18.27-21.57) | 7.12 (5.96-8.48) | <0.01 |
| - ≥95 | 49.23 (47.77 - 50.70) | 42.04 (40.12-43.99) | 34.92 (33.04-36.84) | 23.04 (21.42-24.74) |  |
| Fruit serving, per week | | | | | |
| - < 6 | 36.06 (34.70 - 37.45) | 56.00 (53.71-58.25) | 28.84 (26.8-30.96) | 15.17 (13.63-16.86) | 0.21 |
| - ≥ 6 to <15 | 43.20 (41.73 - 44.69) | 58.55 (56.21-60.85) | 25.98 (24.03-28.03) | 15.47 (13.8-17.29) |  |
| - ≥15 | 20.73 (19.60 - 21.92) | 59.2 (56.09-62.24) | 27.27 (24.57-30.16) | 13.53 (11.48-15.87) |  |
| Vegetables serving, per week | | | | | |
| - <3 | 31.26 (29.93 - 32.62) | 55.18 (52.6-57.74) | 28.06 (25.8-30.43) | 16.76 (14.81-18.91) | 0.07 |
| - ≥ 3 to <9 | 35.26 (33.85 - 36.70) | 57.89 (55.38-60.36) | 27.68 (25.55-29.91) | 14.44 (12.72-16.34) |  |
| - ≥9 | 33.49 (32.12 - 34.88) | 60.04 (57.59-62.44) | 26.14 (24.01-28.38) | 13.82 (12.29-15.52) |  |
| Nuts intake |  |  |  |  |  |
| - <3 times per month | 62.47 (61.03 - 63.88) | 53.47 (51.62-55.32) | 28.90 (27.26-30.59) | 17.63 (16.2-19.16) | <0.01 |
| - 1-6 times a week | 31.76 (30.40 - 33.16) | 65.30 (62.85-67.68) | 24.83 (22.71-27.07) | 9.87 (8.57-11.34) |  |
| - >1 times a day | 5.77 (5.14 - 6.46) | 62.71 (57.11-67.99) | 23.29 (18.86-28.39) | 14.00 (10.82-17.93) |  |

| Table S1-continued: Demographic characteristics of women according to the number of cardiometabolic morbidities; Iran STEPS-2021 | | | | | |
| --- | --- | --- | --- | --- | --- |
| Socio-demographic factors |  | **Number of cardiometabolic morbidities** | | | **p-value** |
|  | **Total (n=9189)** | **0 (n=5091)** | **1 (n=2575)** | **≥2 (n=1523)** |  |
| Wealth index | | | | | |
| - 1st quintile | 19.70 (18.62 - 20.83) | 53.59 (50.58-56.57) | 28.84 (26.34-31.48) | 17.57 (15.61-19.72) | 0.05 |
| - 2nd quintile | 21.74 (20.50 - 23.04) | 54.36 (50.99-57.69) | 28.41 (25.52-31.48) | 17.23 (14.75-20.04) |  |
| - 3rd quintile | 18.47 (17.46 - 19.52) | 57.92 (54.98-60.8) | 27.26 (24.76-29.91) | 14.82 (12.85-17.04) |  |
| - 4th quintile | 19.73 (18.62 - 20.89) | 58.26 (55.02-61.42) | 28.04 (25.18-31.09) | 13.70 (11.51-16.25) |  |
| - 5th quintile (highest) | 20.35 (19.09 - 21.68) | 64.82 (61.19-68.28) | 23.85 (20.85-27.13) | 11.33 (9.09-14.04) |  |
| Family history MI/STROKE | | | | | |
| - Yes | 14.77 (13.74 - 15.86) | 48.85 (44.98-52.74) | 31.50 (28.12-35.09) | 19.65 (16.4-23.35) | <0.01 |
| - No | 85.23 (84.14 - 86.26) | 59.31 (57.75-60.84) | 26.55 (25.19-27.95) | 14.15 (13.11-15.25) |  |
| Family history diabetes |  |  |  |  |  |
| - Yes | 34.08 (32.73 - 35.46) | 48.32 (45.93-50.73) | 29.70 (27.48-32.02) | 21.97 (19.96-24.13) | <0.01 |
| - No | 65.92 (64.54 - 67.27) | 62.64 (60.88-64.37) | 26.03 (24.51-27.6) | 11.33 (10.23-12.53) |  |
| Hospitalized Covid-19 | | | | | |
| - Yes | 1.15 (0.89 - 1.50) | 34.88 (23.83-47.83) | 30.62 (19.02-45.33) | 34.50 (23.7-47.19) | <0.01 |
| - No | 98.85 (98.50 - 99.11) | 58.03 (56.57-59.48) | 27.24 (25.97-28.55) | 14.73 (13.7-15.82) |  |
| Age, years | 46.69 (46.31 - 47.07) | 40.86 (40.43-47.83) | 52.54 (51.92- 53.17) | 58.81 (58.05- 59.56) | <0.01 |
| BMI, kg/m2 | 28.32 (28.18 - 28.47) | 27.13 (26.96–27.30) | 29.56 (29.26-29.86) | 30.64 (30.27–31.02) | <0.01 |
| WC, cm | 94.40 (94.03 - 94.77) | 90.59 (90.15–91.03) | 98.23 (97.53-98.92) | 102.23 (101.34-103.12) | <0.01 |
| SBP, mmHg | 125.91 (125.39 - 126.44) | 115.58 (115.22-115.94) | 138.16 (137.13-139.21) | 143.52 (142.09-144.96) | <0.01 |
| DBP, mmHg | 77.92 (77.69 - 78.25) | 73.37 (73.07- 73.68) | 84.25 (83.58- 84.92) | 83.95 (83.12- 84.79) | <0.01 |
| FBS, mg/dl | 104.70 (103.82-105.58) | 93.39 (93.06–93.73) | 108.61 (106.73-110.48) | 144.11 (140.44-147.77) | <0.01 |
| HbA1c, % | 6.00 (5.97 - 6.03) | 5.53 (5.52–5.54) | 6.17 (6.10-6.24) | 7.52 (7.40-7.64) | <0.01 |
| - Abbreviations: BMI: body mass index; WC: waist circumference; SBP: systolic blood pressure; DBP: diastolic blood pressure; FBS: fasting blood glucose; HbA1c: hemoglobin A1c. - P values < 0.05 considered statistically significant and bolded | | | | | |

| Table S2: Demographic characteristics of men according to the number of cardiometabolic morbidities; Iran STEPS-2021 | | | | | |
| --- | --- | --- | --- | --- | --- |
| Socio-demographic factors |  | **Number of cardiometabolic morbidities** | | | **p-value** |
|  | **Total (n=7264)** | **0 (n=4044)** | **1 (n=2124)** | **≥2 (n=1096)** |  |
| Age groups, years | | | | | |
| - 25-35 | 21.28 (19.90 - 22.73) | 83.51 (79.94-86.55) | 15.47 (12.48-19.01) | 1.02 (0.50-2.05) | <0.01 |
| - 35-45 | 24.93 (23.51 - 26.41) | 73.79 (70.72-76.63) | 22.74 (20.08-25.64) | 3.47 (2.35-5.11) |  |
| - 45-55 | 21.76 (20.48 - 23.10) | 55.53 (52.18-58.83) | 31.44 (28.48-34.56) | 13.03 (10.74-15.73) |  |
| - 55-65 | 19.98 (18.70 - 21.32) | 33.80 (30.54-37.21) | 38.40 (34.84-42.09) | 27.81 (24.68-31.16) |  |
| - 65-75 | 12.05 (11.00 - 13.19) | 27.50 (23.04-32.46) | 36.75 (32.29-41.46) | 35.75 (31.38-40.37) |  |
| Area | | | | | |
| - Urban | 74.91 (73.69 - 76.08) | 57.03 (54.97-59.06) | 28.08 (26.23-30) | 14.90 (13.53-16.38) | <0.01 |
| - Rural | 25.09 (23.92 - 26.31) | 62.16 (59.78-64.48) | 27.38 (25.29-29.57) | 10.46 (9.03-12.10) |  |
| Basic health insurance | | | | | |
| - Yes | 89.34 (88.25 - 90.35) | 56.95 (55.2-58.68) | 28.3 (26.73-29.93) | 14.75 (13.55-16.03) | <0.01 |
| - No | 10.66 (9.65 - 11.75) | 69.74 (64.87-74.20) | 24.56 (20.38-29.28) | 5.70 (4.01-8.06) |  |
| Job | | | | | <0.01 |
| - Unemployed | 6.99 (6.28 - 7.765) | 55.93 (50.63-61.10) | 27.49 (23.24-32.20) | 16.58 (13.32-20.45) |  |
| - Employed | 73.28 (71.8 - 74.72) | 65.51 (63.62-67.35) | 26.25 (24.54-28.03) | 8.24 (7.26-9.33) |  |
| - Unpaid works | 1.90 (1.527 - 2.37) | 60.44 (48.89-70.93) | 31.27 (21.29-43.35) | 8.230 (4.43-15.01) |  |
| - Retired | 17.83 (16.55 - 19.18) | 29.44 (25.92-33.24) | 34.50 (30.68-38.53) | 36.06 (32.20-40.10) |  |
| Marital status | | | | | |
| - Single | 9.56 (8.526 - 10.69) | 79.60 (74.68-83.77) | 17.36 (13.58-21.93) | 3.04 (1.49-6.11) | <0.01 |
| - Married | 88.36 (87.09 - 89.52) | 56.33 (54.6-58.04) | 28.63 (27.09-30.23) | 15.04 (13.83-16.33) |  |
| - Divorced/widow | 2.09 (1.54 - 2.825) | 45.00 (31.12-59.67) | 45.23 (30.04-61.36) | 9.79 (5.72-16.27) |  |
| Education levels, years | | | | | |
| - 0 | 7.46 (6.78 - 8.21) | 39.6 (34.89-44.52) | 35.83 (31.52-40.37) | 24.57 (20.38-29.30) | <0.01 |
| - 1 to <7 | 22.86 (21.62 - 24.14) | 48.54 (45.56-51.53) | 30.96 (28.37-33.66) | 20.50 (18.06-23.18) |  |
| - ≥7 to <12 | 23.20 (21.90 - 24.56) | 62.74 (59.55-65.82) | 28.01 (25.14-31.08) | 9.25 (7.61-11.19) |  |
| - ≥12 | 46.48 (44.80 - 48.17) | 63.91 (61.19-66.56) | 25.07 (22.66-27.65) | 11.01 (9.43-12.82) |  |
| physical activity | | | | | |
| - <1500 min/week | 22.81 (21.54 - 24.13) | 66.65 (63.60-69.56) | 23.88 (21.36-26.60) | 9.47 (7.58-11.76) | <0.01 |
| - ≥1500 min/week | 77.19 (75.87 - 78.46) | 55.85 (53.91-57.78) | 29.09 (27.33-30.92) | 15.06 (13.77-16.44) |  |

| Table S2-continued: Demographic characteristics of men according to the number of cardiometabolic morbidities; Iran STEPS-2021 | | | | | |
| --- | --- | --- | --- | --- | --- |
| Socio-demographic factors |  | **Number of cardiometabolic morbidities** | | | **p-value** |
|  | **Total (n=7264)** | **0 (n=4044)** | **1 (n=2124)** | **≥2 (n=1096)** |  |
| Smoking status | | | | | |
| - Never | 64.01 (62.37 - 65.61) | 57.69 (55.68-59.67) | 28.50 (26.71-30.35) | 13.82 (12.50-15.25) | <0.01 |
| - Past smoker | 9.17 (8.29 - 10.14) | 49.34 (44.12-54.58) | 31.87 (26.87-37.32) | 18.79 (15.12-23.09) |  |
| - Current smoker | 26.82 (25.31 - 28.38) | 62.88 (59.44-66.19) | 25.13 (22.16-28.35) | 12.00 (9.88-14.47) |  |
| Ever alcohol use | | | | | |
| - Yes | 14.26 (13.03 - 15.60) | 61.79 (56.65-66.69) | 25.83 (21.36-30.88) | 12.37 (9.37-16.17) | 0.34 |
| - No | 85.74 (84.40 - 86.97) | 57.74 (56.00-59.45) | 28.25 (26.70-29.85) | 14.02 (12.86-15.26) |  |
| BMI categories, kg/m2 | | | | | |
| - <25 | 39.98 (38.39 - 41.60) | 70.54 (68.21-72.77) | 21.14 (19.28-23.13) | 8.32(6.86-10.06) | <0.01 |
| - ≥25 to <30 | 41.46 (39.82 - 43.12) | 54.8 (52.15-57.42) | 30.62 (28.21-33.14) | 14.58 (12.94-16.40) |  |
| - ≥30 | 18.56 (17.28 - 19.90) | 39.83 (36.15-43.63) | 36.4 (32.52-40.48) | 23.76 (20.69-27.13) |  |
| WC, cm | | | | | |
| - <95 | 48.74 (47.08 - 50.40) | 70.39 (68.21-72.48) | 22.46 (20.58-24.47) | 7.15 (5.97-8.53) | <0.01 |
| - ≥95 | 51.26 (49.60 - 52.92) | 46.83 (44.44-49.24) | 33.07 (30.84-35.38) | 20.09 (18.32-21.99) |  |
| Fruit serving, per week | | | | | |
| - < 6 | 37.13 (35.54 - 38.74) | 58.71 (55.97-61.40) | 28.89 (26.40-31.51) | 12.40 (10.69-14.33) | 0.39 |
| - ≥ 6 to <15 | 40.31 (38.68 - 41.97) | 57.55 (54.88-60.17) | 27.66 (25.31-30.15) | 14.79 (13.09-16.66) |  |
| - ≥15 | 22.56 (21.25 - 23.93) | 59.03 (55.71-62.26) | 26.70 (23.92-29.68) | 14.27 (11.95-16.95) |  |
| Vegetables serving, per week | | | | | |
| - <3 | 34.89 (33.31 - 36.51) | 56.39 (53.49-59.24) | 29.61 (26.95-32.42) | 14.00 (12.11-16.14) | 0.14 |
| - ≥ 3 to <9 | 33.92 (32.38 - 35.50) | 60.08 (57.31-62.79) | 25.42 (23.07-27.92) | 14.50 (12.63-16.61) |  |
| - ≥9 | 31.19 (29.67 - 32.75) | 58.55 (55.63-61.41) | 28.70 (26.13-31.42) | 12.75 (11.01-14.73) |  |
| Nuts intake |  |  |  |  |  |
| - <3 times per month | 60.98 (59.35 - 62.59) | 54.92 (52.79-57.03) | 29.11 (27.20-31.10) | 15.97 (14.43-17.63) | <0.01 |
| - 1-6 times a week | 33.56 (31.99 - 35.16) | 63.79 (60.94-66.55) | 25.76 (23.21-28.49) | 10.45 (8.976-12.12) |  |
| - >1 times a day | 5.46 (4.83 - 6.16) | 62.54 (56.62-68.10) | 27.56 (22.71-33.02) | 9.90 (6.975-13.87) |  |

| Table S2-continued: Demographic characteristics of men according to the number of cardiometabolic morbidities; Iran STEPS-2021 | | | | | |
| --- | --- | --- | --- | --- | --- |
| Socio-demographic factors |  | **Number of cardiometabolic morbidities** | | | **p-value** |
|  | **Total (n=7264)** | **0 (n=4044)** | **1 (n=2124)** | **≥2 (n=1096)** |  |
| Wealth index | | | | | |
| - 1st quintile | 16.68 (15.54 - 17.88) | 61.33 (57.64-64.89) | 26.06 (22.94-29.43) | 12.61 (10.42-15.19) | 0.09 |
| - 2nd quintile | 17.84 (16.62 - 19.13) | 58.87 (54.98-62.64) | 26.07 (22.73-29.71) | 15.07 (12.49-18.06) |  |
| - 3rd quintile | 20.30 (19.12 - 21.54) | 56.74 (53.55-59.87) | 29.80 (27.03-32.73) | 13.46 (11.40-15.82) |  |
| - 4th quintile | 21.71 (20.45 - 23.01) | 60.34 (57.19-63.41) | 25.07 (22.56-27.75) | 14.59 (12.37-17.13) |  |
| - 5th quintile (highest) | 23.47 (21.88 - 25.14) | 55.24 (51.01-59.40) | 31.59 (27.65-35.80) | 13.17 (10.67-16.16) |  |
| Family history MI/STROKE | | | | | |
| - Yes | 11.50 (10.45 - 12.64) | 49.33 (44.27-54.40) | 30.52(26.15-35.27) | 20.15 (16.53-24.34) | <0.01 |
| - No | 88.50 (87.36 - 89.55) | 59.48 (57.73-61.21) | 27.56(25.99-29.19) | 12.96 (11.82-14.18) |  |
| Family history diabetes | | | | | |
| - Yes | 29.72 (28.26 - 31.22) | 48.44 (45.54-51.36) | 30.13 (27.53-32.85) | 21.43 (19.10-23.96) | <0.01 |
| - No | 70.28 (68.78 - 71.74) | 62.49 (60.50-64.44) | 26.96 (25.17-28.83) | 10.55 (9.39-11.83) |  |
| Hospitalized Covid-19 | | | | | |
| - Yes | 0.92 (0.70 - 1.21) | 53.01 (39.59-66.01) | 13.47 (7.678-22.56) | 33.52 (22.45-46.76) | <0.01 |
| - No | 99.08 (98.79 - 99.30) | 58.36 (56.7-60.01) | 28.04 (26.54-29.58) | 13.60 (12.50-14.78) |  |
| Age, years | 47.16 (46.71 - 47.6) | 42.39 (41.87-42.91) | 51.34 (50.50-52.18) | 59.04 (58.17-59.91) | <0.01 |
| BMI, kg/m2 | 26.30 (26.16 - 26.44) | 25.39 (25.22-25.56) | 27.31 (27.05-27.57) | 28.11 (27.73-28.50) | <0.01 |
| WC, cm | 94.55 (94.10 - 95.01) | 91.91 (91.37-92.40) | 97.03 (96.21-97.85) | 100.82 (100.00-102.36) | <0.01 |
| SBP, mmHg | 127.31 (126.76 - 127.85) | 118.70 (118.31-119.09) | 137.62 (136.52-138.71) | 143.05 (141.47-144.63) | <0.01 |
| DBP, mmHg | 79.34 (78.96 - 79.72) | 74.81 (74.50-75.14) | 85.91 (85.11-86.73) | 85.32 (84.34-86.29) | <0.01 |
| FBS, mg/dl | 104.96 (103.80-106.13) | 94.55 (94.15-94.95) | 110.01 (107.3-112.72) | 143.09 (138.02-148.17) | <0.01 |
| HbA1c, % | 6.0 (5.96 - 6.04) | 5.58 (5.56-5.59) | 6.17 (6.07-6.26) | 7.47 (7.31-7.36) | <0.01 |
| - Abbreviations: BMI: body mass index; WC: waist circumference; SBP: systolic blood pressure; DBP: diastolic blood pressure; FBS: fasting blood glucose; HbA1c: hemoglobin A1c. - P values < 0.05 considered statistically significant and bolded | | | | | |

**Figure S1**
